# Transcriptomics Evidence for Common Pathways in Human Major Depressive Disorder and Glioblastoma

**DOI:** 10.3390/ijms19010234

**Published:** 2018-01-12

**Authors:** Yongfang Xie, Ling Wang, Zengyan Xie, Chuisheng Zeng, Kunxian Shu

**Affiliations:** Institute of Bioinformatics, Chongqing University of Posts and Telecommunications, Chongqing 400065, China; xieyf@cqupt.edu.cn (Y.X.); xiezy@cqupt.edu.cn (Z.X.); zengcs@cqupt.edu.cn (C.Z.)

**Keywords:** major depressive disorder, glioblastoma, differentially expressed genes, transcriptomics, common pathway

## Abstract

Depression as a common complication of brain tumors. Is there a possible common pathogenesis for depression and glioma? The most serious major depressive disorder (MDD) and glioblastoma (GBM) in both diseases are studied, to explore the common pathogenesis between the two diseases. In this article, we first rely on transcriptome data to obtain reliable and useful differentially expressed genes (DEGs) by differential expression analysis. Then, we used the transcriptomics of DEGs to find out and analyze the common pathway of MDD and GBM from three directions. Finally, we determine the important biological pathways that are common to MDD and GBM by statistical knowledge. Our findings provide the first direct transcriptomic evidence that common pathway in two diseases for the common pathogenesis of the human MDD and GBM. Our results provide a new reference methods and values for the study of the pathogenesis of depression and glioblastoma.

## 1. Introduction

Glioma is the most common tumor in the central nervous system, mostly occurring in the brain, and the diagnosis and treatment of glioma are incomplete, inaccurate, and easily reappeared. The current study [1,2] shows that most patients with glioma can get better diagnosis and treatment, but the diagnosis and treatment results are still unsatisfactory, even with depression. Moreover, the pathogenesis of depression is still unknown, which seriously hinders the prevention, diagnosis, and treatment of depression. Therefore, depression is one of the major causes of global disability and has considerable risks in patients with gliomas. Depression has become a common complication of brain tumors [3], and has become the first clinical manifestation of gliomas in clinical diagnosis. Seddighi et al.’s studies have shown that depressive symptoms are shown to be common signs in patients with brain tumors [4]. They suggest that statistical analysis of the deterioration of psychiatric symptoms mentioned in the later stages of tumorigenesis is not feasible due to the high variability of tumor staging. Glioblastoma (GBM) is a rare malignant tumor that arises from astrocytes—the star-shaped cells that make up the “glue-like” or supportive tissue of the brain and is the most malignant glioma in astrocytic tumors. Despite all therapeutic efforts, GBM remains largely incurable.

**Figure 1:**
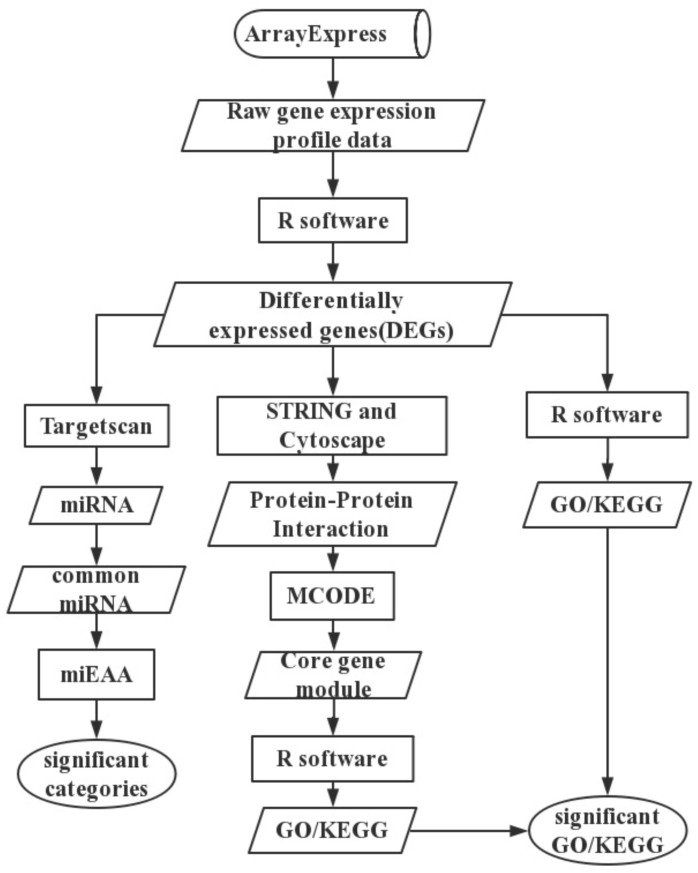
The flowchart of the research program. Cylinder: the database; Rectangle: method or software; Parallelogram: data or result; Ellipse indicates the finally result.

Aiming at this problem, this study uses GBM and major depressive disorder (MDD) as the research object to study the overlapping genes, miRNA, biological pathways, and so on. Is the statistical analysis of the correlation between MDD and GBM feasible? With the implementation of the human genome project (HGP), the Human Proteome Project (HPP), and the Human Connectome Project (HCP), more and more ion channels, cytokines, growth factors, neurotransmitters and neurotransmitter receptors, enzymes, other proteins, and miRNA associated with the development of depression and glioblastoma diseases, have been identified and validated [5]. Therefore, it is feasible to analyze the correlation between MDD and GBM by the method of omics. But, few new and effective treatments appear. At present, RNA interference has enormous therapeutic potential for two diseases. Therefore, it is the best way to explore the pathogenesis of the disease through transcriptome data. This study designs a set of transcriptomics in three directions to study the common pathways of disease programs, the flowchart can be found in Figure 1. The process is mainly to analyze the function of RNA in coding region and non-coding region. It mainly divided into three parts. (1) The differentially expressed genes (DEGs) were screened from the gene expression profile data by R software and its corresponding expansion kit [6,7,8,9], and the gene ontology (GO), Kyoto Encyclopedia of Genes and Genomes(KEGG) results were significantly correlated with functional enrichment analysis; (2) Using the STRING [10] and Cytoscape [11] tools to construct the protein—protein interaction (PPI) network, the core gene module was excavated by MCODE [12] algorithm, and the GO and KEGG results of MDD and GBM overlap were obtained by functional enrichment analysis; and (3) Targetscan [13] tool was used to predict the miRNA of differentially expressed genes in two diseases, and to enrich, analyze, and annotate the overlapped miRNA in two diseases by miEAA [14]. This study finds from another direction the pathogenesis of the disease. It is hoped that these findings will provide new ideas for the diagnosis and treatment of MDD and GBM.

## 2. Results

### 2.1. The Common Co-Occurrence Gene by Text-Mining

Through COREMINE platform text mining tools, MDD and 1826 genes have co-occurrence relationship, Glioma and 1826 genes have co-occurrence relationship, GBM and 4510 genes have co-occurrence relationship. Among them, 57% of MDD co-occurrence genes and 23.1% of GBM co-occurrence genes were identified as common genes, with a total of 1041 genes (Table 1). Besides, it is shared with 78 co-occurrence GO cellular component (CC), 317 co-occurrence GO biology process (BP), and 52 co-occurrence GO molecular function(MF) betweenthe two diseases. Our finds speculated that may have common biological pathways or the occurrence of the same mutation between MDD and GBM.

### 2.2. Differentially Expressed Genes

After the DEGs was screened out, the DEGs of different platforms of the same disease were combined as the final DEGs of the disease. There are 463 DEGs (*p*-value < 0.01) significantly associated with MDD, and 823 DEGs (*p*-value < 0.05 and fold change ≥ 4) were significantly associated with GBM. A simple statistical analysis of DEGs revealed that a total of 27 genes were not only significantly associated with MDD but also closely related to GBM. It was found that five genes (*GRK3, SHANK3, EGR4, CRH, GNB5*) in these 27 genes are down-regulated genes, and six genes (*IGF2BP3, MGP, LOX, KCNE4, DLGAP5, MS4A7*) are up-regulated genes.

Statistics were found through literature mining, in 463 MDD DEGs, 80 genes have been reported related to MDD, there are 201 genes associated with depression; in 823 GBM DEGs, 452 genes are reported with GBM; 27 DEGs overlap in MDD and GBM, eight genes has been reported related to MDD, 14 genes have been reported related to GBM. Moreover, four genes in the reported gene are associated with both MDD and GBM. The four genes are *LOX, NPY1R, SHANK3, VEGFA*. The study finds that LOX expression and activity increased positively correlated with GBM [15]. MDD treatment of electroconvulsive shock (ECS) can be induced by activity-dependent induction of genes (FOX) that are associated with plasticity of the brain, such as neuronal signaling-induced neurogenesis and tissue remodeling [16]. Berent et al. found that higher VEGFA concentrations may have antidepressant effects [17]. Therefore, VEGFA may play a potentially important role in the pathogenesis of MDD. However, Stefano et al. suggest that VEGFA triggers an angiogenic response and promotes GBM vascular growth [18]. There are indications that have been screened for differentially expressed genes that are reliable. We can carry out the next step of the functional analysis.

### 2.3. Functional Enrichment of DEGs

**Figure 2:**
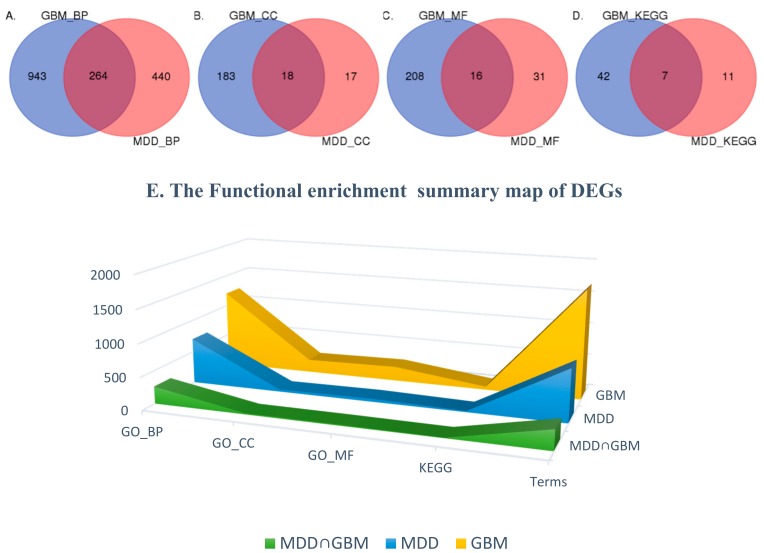
Differentially Expressed Genes Enrichment Venn Diagram and Its 3D Area Map. Figure (**A**–**D**) indicate similarities and differences in the functional enrichment results of two diseases. They are GO_BP, GO_CC, GO_MF, KEGG. In Figure (**E**), the Yellow: GBM enrichment results; blue: MDD functional enrichment results; green: MDD and GBM common enrichment results. MDD: Major Depressive Disorder; GBM: glioblastoma; BP: Biology Process; CC: Cellular Component; MF: Molecular Function.

The R tool is used to analyze and enrich the DEGs. DEGs in MDD were significantly enriched in 804 terms (count ≥ 2 and *p*-value < 0.05), including 704 GO biology process terms, 35 GO cellular component terms, 47 GO molecular function terms, and 18 KEGG pathway terms. DEGs in GBM are significantly enriched in 1681 terms, involving 1207 GO biology process terms, 201 GO cellular component terms, 224 GO molecular function terms, and 48 KEGG pathway terms. These results show that MDD and GBM have 264 BP, 18 CC, 16 MF functional annotations overlap in GO, and seven biological pathways overlap in KEGG. Figure 2 shows the same functional enrichment results for the Wein diagram and its proportion in both diseases. It can be found that the enrichment of the two diseases has some common ground. The same GO or KEGG of the two diseases is approximately 1/3 of the MDD functional enrichment results, approximately 1/10 of the GBM functional enrichment.

The 1680 GBM function enrichment results and 804 MDD function enrichment results are summarized, and 305 common data of MDD and GBM are extracted. Pearson’s method was used to calculate the correlation coefficient of the respective differentially expressed genes of MDD and GBM in the common data. Finally, calculated the correlation coefficient between the two is 0.9525328, close to 1, the relevance and high. That is, even though only 27 of the two diseases overlap, almost completely different differentially expressed genes. There is also an extremely high correlation in this common functional enrichment data, suggesting that MDD may also have some relevance to the underlying pathogenesis of GBM. Of course, we also functionally enrich 27 co-differentially expressed genes and obtain three significant KEGG pathways.

### 2.4. Protein-Protein Interaction Network of DEGs

**Figure 3:**
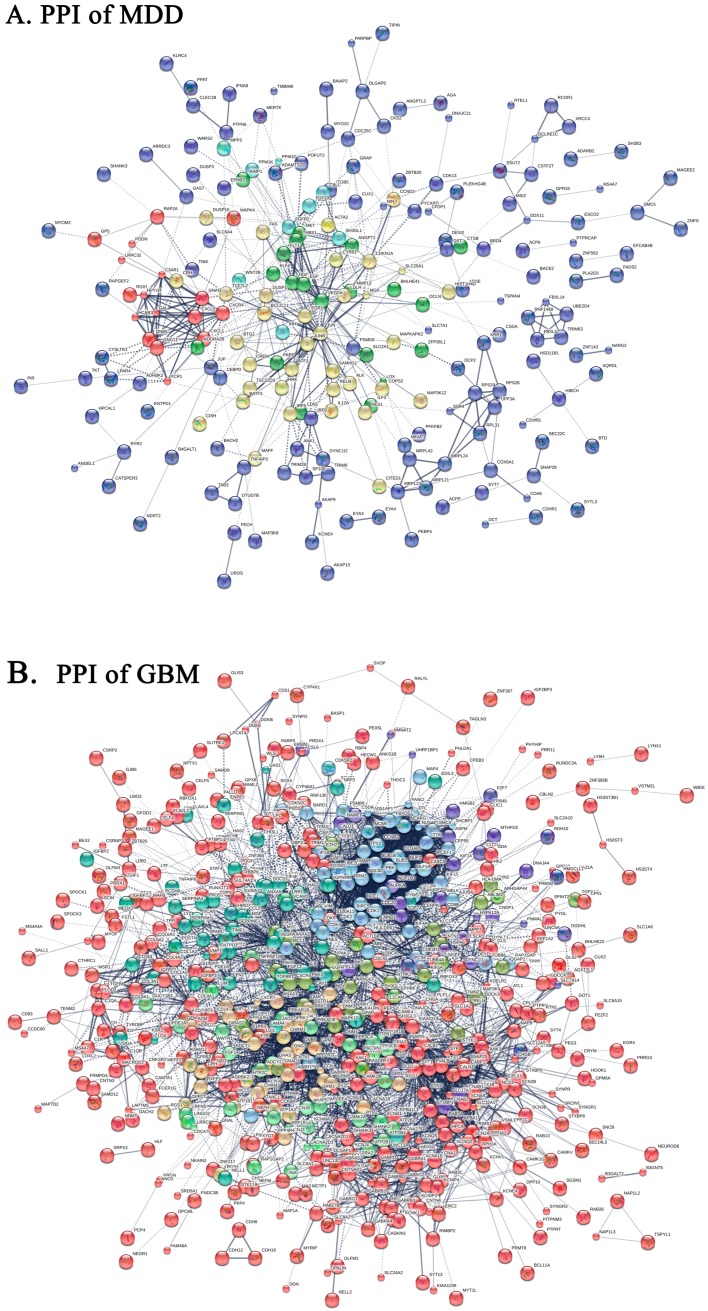
The protein-protein interaction network of MDD and GBM. (**A**) The protein—protein interaction (PPI) of MDD. (**B**) The PPI of GBM. Nodes of the same color represent proteins that are aggregated into the same class; large nodes indicate that the three-dimensional structure of the protein is known and that the small nodes are unknown; the line represents the interaction between proteins; there are seven kinds of relationship. Red, fusion gene; Green, adjacent interaction; Blue, coexistence relationship; Purple, experimental study of validation interactions; Yellow, literature digging to the interaction; and, Light blue, the database included interaction; Black, shared expression. MDD, Major Depressive Disorder. GBM, glioblastoma.

In this study, we use the STRING online tool to construct the PPI of 402 nodes and 512 sides for MDD, as well as PPI of 794 nodes and 4443 sides for GBM (Figure 3). The Cytoscape tools are used to build the interaction of MDD and GBM PPI. Based on PPI (the elimination of independent protein), 74 and 64 HUB genes (Table 2) (Betweenness Centrality (BC) ≥ 0.01, degree ≥ 2) were closely related to MDD and GBM. Among the key genes in MDD and GBM, four genes exist together. Namely, *CXCR4, VEGFA, MGP, GNB5*, and *MGP* genes are down-regulated in two diseases, while *GNB5* gene is co-up-regulated.

**Figure 4:**
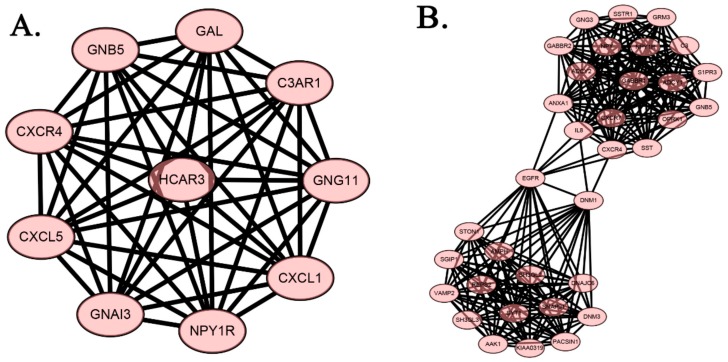
PPI of the core gene module. (**A**) The PPI of the most significant core module about MDD. (**B**) The PPI of the secondary core gene module about GBM. Node: protein; connection: the interaction between proteins. MDD, Major Depressive Disorder. GBM, glioblastoma.

MCODE algorithm is used to cluster MDD and GBM PPI, respectively. The PPI of MDD can be clustered into 11 categories, and the GBM of PPI is clustered into 20 categories (Table S1). In the MDD’s 11 core gene module, the functional enrichment of the most significant class (Figure 4 and Table 3) found that GABAergic synapse, Serotonergic synapse, Cholinergic synapse, Glutamatergic synapse, Dopaminergic synapse, and Morphine addiction affect the development of depression. In the GBM’s 20 core gene module, the functional enrichment of the second significant group (Figure 4 and Table 3) also found that GABAergic synapse, Serotonergic synapse, Cholinergic synapse, Glutamatergic synapse, and Morphine addiction, were associated with the development of GBM. Therefore, the two core gene modules with high significance and overlapping biological pathways are regarded as the significant core gene modules of disease. Moreover, there are two common key genes in the core gene module, that is CXCR4 and GNB5. The accumulation of two significant core gene modules revealed that 10 biological pathways overlap, accounting for 71.4% of MDD enrichment, and 50% of GBM enrichment. The two core gene modules with higher saliency and overlapping biological pathways as the significant core gene modules of the disease. In addition, there are two overlapping key genes in significant core gene modules—CXCR4 and GNB5, accounting for half of the overlapping key genes of both diseases. These two significant core gene modules may play an important role in the underlying pathogenesis of MDD or GBM. Ten common KEGG biological pathways of significant core gene modules are the likely common pathways of MDD and GBM.

### 2.5. miRNA

**Figure 5:**
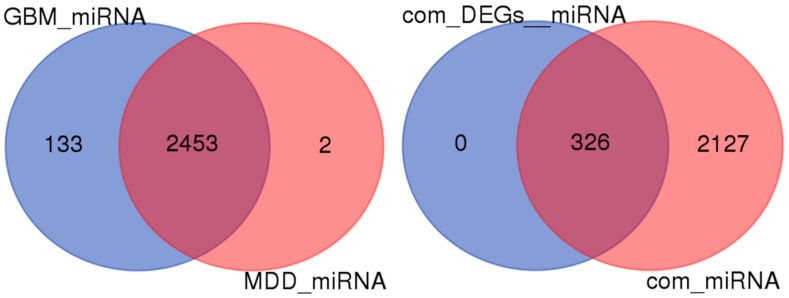
The predicted miRNA Venn Diagram. GBM_miRNA: the miRNAs predicted by GBM-related DEGs; MDD_miRNA: the miRNAs predicted by MDD-related DEGs; com_DEGs_miRNA: the miRNAs predicted by common 27 DEGs; com_miRNA: common miRNA between GBM_miRNA and MDD_miRNA.

This study uses the TargetScanHuman tool to predict the related miRNAs of DEGs (mRNA). After a series of screening criteria, 18,656 pairs for MDD mRNA—miRNA, 52,413 pairs for GBM mRNA—miRNA are obtained. Data analysis of MDD reveals that there are 370 different mRNA corresponding to 2455 different miRNA. Data analysis of GBM reveals that there are 754 different mRNA, corresponding to 2586 different miRNA. Figure 5 intuitively shows the miRNA of two diseases, overlapping up to 2453. In the figure, 99.9% of the miRNAs predicted by MDD-related DEGs are the same as the miRNA predicted by GBM. Moreover, the predicted miRNAs of the 27 common DEGs to the two diseases completely overlap with the common miRNAs predicted by the two diseases.

### 2.6. Functional Enrichment of Common DEGs and Common miRNA

Through the enrichment of 27 common DEGs in MDD and GBM, three KEGG biological pathways are enriched, which are Cytokine—cytokine receptor interaction, Chemokine signaling pathway, Bladder cancer. There are 128 GO biology process (BP) terms, two GO cellular component (CC) terms, three GO molecular function (MF) terms are significantly enriched (Table S2). The results showed that the cellular components of common DEGs in the two diseases are related to the activity of transcription factors. Its molecular function is related to the extracellular matrix, and its biological process is mainly involved in the regulation of multicellular biological processes, the regulation of ion transport, the regulation of growth and development, and the response to some stimuli, and so on.

The common miRNAs are analyzed by miEAA, and their enrichment and annotation results are shown in Table 2, where the enrichment results of GO are not fully shown. These miRNAs are closely related to the expression of CD3, CD14, CD19, and CD56 in four immune cells, indicating that both MDD and GBM can cause immune system disorders. 2453 common miRNAs have 102 miRNAs located on chromosome 7, indicating that chromosome 7 is not only associated with mental illness, such as depression and schizophrenia, but are also closely related to the pathogenesis of glioblastoma [19,20]. The results show that there are seven biological pathways (Table 4), 356 gene ontologies are enriched. In the 7 pathways, two are related to amino acid metabolism, two are related to carbohydrate metabolism, two are related to mRNA processing, and one is the Notch signaling pathway that affects multiple processes that occur in cells.

## 3. Discussion

To comprehensively and accurately identify the pathogenesis of MDD and GBM, all available transcript data for both of the diseases are downloaded. The purpose is to horizontally merge large amounts of transcriptome data to expand the sample size and obtain a larger sample size dataset. Functional analysis of the differential expression genes of the two diseases is carried out from three aspects.

From the perspective of coding genes, MDD and GBM differentially expressed genes are enriched in seven common biological pathways, namely Melanoma, Pathways in cancer, mitogen-activated protein kinase (MAPK) signaling pathway, Endocytosis, p53 signaling, Focal adhesion, Bladder cancer. As a result, it has been found that five common pathways are associated with the development of cancer, suggesting that the two diseases may also be closely related to other diseases, particularly cancer. Due to the complexity of cancer, the five pathways are temporarily serve as the common pathway for the two diseases. At the same time, it can see that the other two common pathways, MAPK signaling pathway and Endocytosis, are reported to be associated with both diseases. The MAPK pathway may be initiated at the cell surface and continue during endosomal sorting, while more recent studies suggest that MAPK signaling is a required element of endocytosis [21]. Li Kai et al. found the disturbance mechanism of MAPK and cell cycle signaling pathway in GBM by bioinformatics analysis [22]. The study has found that the MAPK signaling pathway is impaired in MDD and plays a key role in neuronal plasticity and neurogenesis, and is shown to be stimulated by an antidepressant treatment [23]. It is suggested by the results that MAPK signaling pathways may be one of the common pathways for MDD and GBM. Cytokine—cytokine receptor interaction, Chemokine signaling pathway and Bladder cancer are enriched by MDD and GBM common DEGs. These three biological pathways also belong to the common biological pathway of MDD and GBM.

From the perspective of miRNA, the corresponding miRNAs are predicted by the mRNA of the two diseases, and the number of common miRNAs has been found to be 2453. In addition, various miRNAs have been demonstrated to be either upregulated or downregulated in glioma tumors, and played critical roles in regulating glioblastoma proliferation, migration, and chemosensitivity [24]. Several recent studies have suggested the possible role of miRNAs in synaptic plasticity, neurogenesis, and stress response, all implicated in MDD [25]. Most of these miRNAs are contained in common miRNAs. For example, Hsa-miR-21 is not only involved in the alterations of white matter in depression and alcoholism [26], but it also plays a key role in the pathogenesis of GBM and can be used as a biomarker for the diagnosis and treatment of GBM patients [27]. The miRNAs were found to be closely related to the abnormal expression of CD3, CD14, CD19, and CD56 in immune cells category. CD3 and CD4 are protein mixtures present on the surface of T cells, CD19 is a protein present on the surface of B cells, and CD56 is an affinity binding glycoprotein expressed on the surface of neurons, glial, and skeletal muscle. CD3, CD4, CD19 are related to the immune process, CD56 role in the p59Fyn signaling pathway. Are the common pathogenesis of the two diseases related to the immune system? There is no confirmation here. It can only be said that the pathogenesis of MDD and GBM may be associated with the immune system. In the seven biological pathways enriched by common miRNA, four pathways are metabolically related, two pathways are associated with mRNA processes, and one is Notch signaling pathway. On the one hand, the possible reason is that MDD and GBM patients biochemical environment affects the brain tissue, metabolic changes occur. On the other hand, Irshad et al. have identified the key molecular cluster characteristics of the Notch pathway response in hypoxic GBM and glial cell spheres [28]. Moreover, Ning et al. also determined that differential expression of Notch-associated miRNAs in peripheral blood may be involved in the development of depression [29]. Thus, glycine—serine and threonine metabolism, inositol phosphate metabolism, arginine and proline metabolism, N-acetylglucosamine metabolism, and Notch signaling pathways are also common biological pathways for MDD and GBM.

From the perspective of protein interaction, the significant core gene modules in the MDD and GBM protein interaction networks were enriched to 10 common biological pathways. The discovery of significant core gene modules in protein—protein interaction networks allows for a more accurate and comprehensive understanding of the function of DEGs in disease. The γ-aminobutyric acid (GABA), glutamic acid, and acetylcholine (Ach) are three common amino acid neurotransmitters, which are specific chemicals that act as “messengers” in synaptic transmission. Salvadore et al. confirm that amino-acid neurotransmitter system dysfunction plays a major role in the pathophysiology of major depressive disorder [30]. Panosyan et al. have found that these three neurotransmitters are involved in the metabolic pathways underlying the potential targets of GBM therapy, but the hypothesis that they have a significant antitumor effect on GBM has not been demonstrated [31]. Hence, GABAergic synapse, Cholinergic synapse, and Glutamatergic synapse may be common pathways for MDD and GBM. Retrograde endocannabinoid signaling has been shown to be related to the pathophysiological mechanisms of MDD and GBM [32,33]. Pathways in cancer, Chemokine signaling pathway is also a common pathway when using genomics enrichment. Therefore, Pathways in cancer, Retrograde endocannabinoid signaling, Chemokine signaling pathway may also be a common pathway for MDD and GBM.

## 4. Materials and Methods

### 4.1. Text Mining

In the biomedical field, text mining has been widely used to identify biological terms, such as genes, disease names in the literature, and even reveal the relationship between biological terms. In this study, COREMINE [34] (Available online: http://www.coremine.com/medical/), a medical ontology information retrieval platform, was used to search for key words, such as major depressive disorder and glioblastoma.

### 4.2. Data and Data Preprocessing

The original gene expression profiling data was based on the GPL570 and GPL17027 platform developed by Affymetrix, derived from EBI’s common library database ArrayExpress [35] (Available online: http://www.ebi.uk/ArrayExpress). Including transcriptome data sets of major depressive disorder (excluding bipolar disorder) and glioblastoma. A total of 47 series, 2093 samples of raw data, 11 series, 367 samples were associated with MDD, 36 series, and 1726 samples were associated with GBM (Table S3 for data sources). In this study, we used the RMA (Robust Multichip Average) method in the Affy package of the R tool to normalized the raw data and then obtained the corresponding gene expression matrix.

### 4.3. Differential Expression Analysis

Studies had found that disease is associated with genes, even if only a small change in a subunit in the genome. For example, the duplication or absence of a dose-sensitive gene [36] is associated with disease, including heart disease, cancer, and neuropsychiatric disorders. Therefore, the use of differential expression analysis method to identify the disease-related genes is essential. In this study, the linear regression and classical Bayesian method in the limma package of R language were used to analyzed and screened differentially expressed genes of the two diseases. Since the two diseases do not belong to the same type of disease, the screening criteria for differentially expressed genes use different thresholds. MDD differential expression gene screening criteria were *p*-value threshold of 0.01, GBM differential expression gene screening criteria were *p*-value threshold is 0.05, and the difference in expression was greater than 4.

### 4.4. Functional Enrichment Analysis

Functional enrichment analysis is a method of cross-integration between biology and mathematics, which is the best choice to solve the massive data of gene chip. In this study, we used the GOstats and KEGG.db toolkit in the R language to perform functional enrichment analysis on the significantly differentially expressed genes and select the GO entry with a Count value greater than or equal to 2 and a *p*-value of less than 0.05. At the same time, the KEGG pathway with *p*-value less than 0.05 was selected as the enrichment biological pathway.

### 4.5. Protein—Protein Interaction Network

In this study, the STRING (Available online: http://string-db.org/) database was used to construct the Protein-Protein Interaction (PPI) between proteins encoded by differentially expressed genes. The STRING database is a database that collects protein—protein interactions, gene regulatory relationships, document mining analysis, and protein co-expression analysis, and calculates physical interactions and predicts interaction relationships. The protein interaction threshold was set at 0.4. The protein interaction data obtained from the online STRING database is imported into the Cytoscape software, and the node with the degree greater than 2 and the BC was greater than 0.01 was obtained by using its Network Analysis plug-in tool. The node as a network centre node (Hub). The protein represented by the central node was usually the key protein (Hub gene) [37] with important physiological functions. Then, the MCODE algorithm in Cytoscape was used to further cluster analysis to find the core gene module in the protein interaction network, and to dig the biological function or pathway that was significantly related to the disease.

### 4.6. Predicted miRNAs

Numerous studies have confirmed that alterations of specific microRNAs (miRNAs) levels are closely related to human pathologies [38]. A small number of miRNA biological functions have been elucidated. Thus, miRNAs were predicted by the TargetScanHuman (Available online: http://www.targetscan.org/vert_71/) tool for differentially expressed genes. The standard for screening predicted miRNAs was 8 mer—a (exact match to positions 2–8 of the mature miRNA followed by an “A”) and the percentage of context ++ score (CS) should not be less than 95%. This CS is the cumulative sum of 14 features for a particular site, including the type of site, complementary pairing, minimum distance, length of open reading frame (ORF), conserved target probability (PCT), and so on. To further analyze and explore the pathogenesis of the disease. The miEAA (miRNA Enrichment Analysis and Annotation Tool, Available online: https://ccb-compute2.cs.uni-saarland.de/mieaa_tool/) online tool was used to enrich and annotate the predicted miRNAs by combining the two diseases. The miEAA’s *p*-value adjustment method was error detection rate (false discovery rate, FDR), the category of *p*-value less than 0.1 was significantly related.

## 5. Conclusions

In this article, we first rely on transcriptome data to obtain reliable and useful differentially expressed genes (DEGs) by differential expression analysis. Then, we used the transcriptomics of DEGs to find out and analyze the common pathway of MDD and GBM from three directions. At present, more and more miRNA are the biomarkers of disease, which are related to the pathophysiology of various diseases, including MDD and GBM. However, due to the large number of predicted miRNAs, further studies are needed to find suitable biomarkers for MDD and GBM. Finally, we determine the important biological pathways that are common to MDD and GBM by statistical knowledge. It is worth mentioning that, Chemokine signaling pathway not only found in functional enrichment of coding genes is a common pathways between MDD and GBM, also found in the core gene module of the protein interaction network. Our findings provide the first direct transcriptomic evidence that common pathway in two diseases for the common pathogenesis of the human MDD and GBM. Our results provide a new reference methods and values for the study of the pathogenesis of depression and glioblastoma.

## Figures and Tables

**Table 1:** The results of text-mining in COREMINE platform. MDD: Major Depressive Disorder; GBM: glioblastoma; Related articles: Pubmed search with a concept or expert name to generate a list of articles; BP: Biology Process; CC: Cellular Component; MF: Molecular Function.

Disease	Related Articles	Gene/Protein	Chemical	CC	BP	MF
MDD	34377	1826	3511	110	498	104
GBM	30193	4510	7779	229	834	244
GBM ∩ MDD	4	1041	2248	78	317	52

**Table 2:** MDD and GBM Hub genes.

GBM	MDD
HS6ST3; ZNF385B; VSTM2L; EGFR; VEGFA; TOP2A; CDC42; MYC; IL8; FN1; PRKACB; CD44; CDK1; VIM; GFAP; SYP; PPP3CA; SNCA; STX1A; DNM1; GNAO1; CACNA1B; LPL; PCSK2; PRKCB; SYT1; SNAP25; TUBB4A;DLG2; PVALB; CAMK2A; CALB1; CDK2; CAV1; CXCR4; GNB5; VAMP2; NPY; VCAM1; PRKCE; C3; EZH2; CDC20; SST; GAD2; ITPR1; ADCY2; LUM; TAC1; AURKA; CD163; SYN1; SPARC; BIRC5; GABBR2; ANXA1; MGP; GAD1; TYMS; GNG3; SCN2A; MCL1; CNKSR2; NDE1	JUN; VEGFA; EGF; CXCR4; GNAI3; EGR1; FLT1; CDKN1A; ATF3; CXCL1; GNG11; GNB5; ACTA2; MET; FGFR2; SH3GL1; CXCL5; MGP; THBS1; FAS; IRF5; JUP; RAP2A; TCF7L2; MRPL23; TNFAIP3; CCND3; SLC25A1; MAPK4; BATF3; CD55; CDC25C; MPP3; PPM1D; ILF3; HIST1H4D; CDK13; SSU72; PTPN6; CREM; OCLN; ADORA2B; HIBCH; DYNC1I2; CTSB; MAFF; RYR2; DLGAP5; DCLRE1C; SSR4; ADRBK2; COPS2; COX6A1; LOX; SNAP29; BRD4; DDX11; KRR1; AKAP9; SMC5; ZFP36L1; AIMP1; CFDP1; GAS7; MYO10; GP5; SYT7; ESCO2; MSI2; CLEC1B; FECH; B4GALT1; TKT

**Table 3:** Functional enrichment of significant core gene module—KEGG. MDD, Major Depressive Disorder. GBM, glioblastoma. MDD KEGG: the unique KEGG pathways of significant core gene module of Major Depressive Disorder; GBM KEGG: the unique KEGG pathways of significant core gene module of glioblastoma; Common KEGG: the common KEGG pathways of significant core gene module in Major Depressive Disorder and glioblastoma.

MDD KEGG	GBM KEGG	Common KEGG
Alcoholism	Endocytosis	GABAergic synapse
Pertussis	Gap junction	Cholinergic synapse
Serotonergic synapse	Insulin secretion	Pathways in cancer
Dopaminergic synapse	Salivary secretion	Morphine addiction
	Synaptic vesicle cycle	Circadian entrainment
	Gastric acid secretion	Glutamatergic synapse
	GnRH signaling pathway	cAMP signaling pathway
	Estrogen signaling pathway	Chemokine signaling pathway
	Oxytocin signaling pathway	Retrograde endocannabinoid signaling
	Neuroactive ligand-receptor interaction	Regulation of lipolysis in adipocytes

**Table 4:** Results of the common miRNA enrichment and annotation.

Category	Subcategory	*p*-Value	Observed
Pathways	WP411 mRNA processing	0.060337	145
Pathways	hsa00260 Glycine serine and threonine metabolism	0.060337	30
Pathways	hsa00562 Inositol phosphate metabolism	0.060337	99
Pathways	hsa03040 Spliceosome	0.060337	138
Pathways	hsa00330 Arginine and proline metabolism	0.081737	67
Pathways	hsa04330 Notch signaling pathway	0.081737	94
Pathways	P02756 N acetylglucosamine metabolism	0.095531	14
Immune cells	CD3 expressed	0.008542	205
Immune cells	CD19 expressed	0.029347	182
Immune cells	CD14 expressed	0.040935	235
Immune cells	CD56 expressed	0.055024	252
Chromosomal location	Chromosome 7	0.036298	102
Gene Ontology	GO0042832 defense response to protozoan	0.0626691	11
Gene Ontology	GO0045859 regulation of protein kinase activity	0.0626691	41
Gene Ontology	GO0048304 positive regulation of isotype switching to igg isotypes	0.0626691	11
Gene Ontology	GO0016290 palmitoyl coa hydrolase activity	0.0665877	24
Gene Ontology	GO0044130 negative regulation of growth of symbiont in host	0.0681013	19
Gene Ontology	GO0004439 phosphatidylinositol 4 5 bisphosphate 5 phosphatase activity	0.0753516	16
Gene Ontology	GO0004523 ribonuclease h activity	0.0753516	19
Gene Ontology	GO0031848 protection from non homologous end joining at telomere	0.0753516	16

## Supplementary Materials

Supplementary materials can be found at www.mdpi.com/1422-0067/19/1/234/s1.
