# Supplementary material for: Transcriptomics Evidence for Common Pathways in Human Major Depressive Disorder and Glioblastoma
Source: Int J Mol Sci. 2018 Jan 12;19(1):234. doi: 10.3390/ijms19010234 (PMC5796182; doi:10.3390/ijms19010234)
Supplement: Supplementary file 1 [file ijms-19-00234-s001.pdf]

Supplementary Information

Table S1. Clustering information of protein-protein interaction networks. MDD, Major Depressive Disorder. GBM, glioblastoma. Score, density multiplied by the number of member; Nodes, Number of nodes; Edges, Number of edges.

GBM_Cluster	Score	Nodes	Edges	MDD_Cluster	Score	Nodes	Edges
1	40.244	42	825	1	10	10	45
2	16.97	34	280	2	5	5	10
3	10.973	38	203	3	4.667	7	14
4	7	7	21	4	4.5	5	9
5	5.882	18	50	5	4.5	5	9
6	5.429	15	38	6	4	5	8
7	4.471	35	76	7	3.6	6	9
8	4.375	17	35	8	3.333	4	5
9	4	4	6	9	3	3	3
10	4	5	8	10	3	3	3
11	4	7	12	11	3	3	3
12	3.818	12	21				
13	3.5	5	7				
14	3	3	3				
15	3	3	3				
16	3	3	3				
17	3	3	3				
18	3	3	3				
19	3	3	3				
20	2.952	22	31				

Table S2. The functional enrichment results of common DEGs in MDD and GBM. GO, Gene Ontology: BP, Biology Process; CC, Cellular Component; MF, Molecular Function; KEGG, Kyoto Encyclopedia of Genes and Genomes.

Enriched ID	Term
GOBPID:0044699	single-organism process
GOBPID:0065007	biological regulation
GOBPID:0050789	regulation of biological process
GOBPID:0050794	regulation of cellular process
GOBPID:0019222	regulation of metabolic process
GOBPID:0048518	positive regulation of biological process
GOBPID:0031323	regulation of cellular metabolic process
GOBPID:0060255	regulation of macromolecule metabolic process
GOBPID:0051179	localization
GOBPID:0048519	negative regulation of biological process
GOBPID:0048523	negative regulation of cellular process
GOBPID:0051239	regulation of multicellular organismal process
GOBPID:0048856	anatomical structure development
GOBPID:0051234	establishment of localization
GOBPID:0048522	positive regulation of cellular process
GOBPID:0048731	system development
GOBPID:0065008	regulation of biological quality
GOBPID:0048513	organ development

GOBPID:0009893	positive regulation of metabolic process
GOBPID:0010646	regulation of cell communication
GOBPID:0023051	regulation of signaling
GOBPID:0010033	response to organic substance
GOBPID:0032879	regulation of localization
GOBPID:0051240	positive regulation of multicellular organismal process
GOBPID:0065009	regulation of molecular function
GOBPID:0050793	regulation of developmental process
GOBPID:0051049	regulation of transport
GOBPID:0032268	regulation of cellular protein metabolic process
GOBPID:0071310	cellular response to organic substance
GOBPID:0008219	cell death
GOBPID:0016265	death
GOBPID:0012501	programmed cell death
GOBPID:0006915	apoptotic process
GOBPID:2000026	regulation of multicellular organismal development
GOBPID:0009628	response to abiotic stimulus
GOBPID:0006357	regulation of transcription from RNA polymerase II promoter
GOBPID:0006468	protein phosphorylation
GOBPID:0042592	homeostatic process
GOBPID:0014070	response to organic cyclic compound
GOBPID:0007610	behavior
GOBPID:0032270	positive regulation of cellular protein metabolic process
GOBPID:0055085	transmembrane transport
GOBPID:0007186	G-protein coupled receptor signaling pathway
GOBPID:0022414	reproductive process
GOBPID:0060341	regulation of cellular localization
GOBPID:0010648	negative regulation of cell communication
GOBPID:0023057	negative regulation of signaling
GOBPID:0051094	positive regulation of developmental process
GOBPID:0009611	response to wounding
GOBPID:0048878	chemical homeostasis
GOBPID:0034220	ion transmembrane transport
GOBPID:0009887	organ morphogenesis
GOBPID:0072358	cardiovascular system development
GOBPID:0072359	circulatory system development
GOBPID:0032101	regulation of response to external stimulus
GOBPID:0019725	cellular homeostasis
GOBPID:0042060	wound healing
GOBPID:0043269	regulation of ion transport
GOBPID:0034762	regulation of transmembrane transport
GOBPID:0034765	regulation of ion transmembrane transport
GOBPID:0000003	reproduction
GOBPID:0032940	secretion by cell
GOBPID:0044703	multi-organism reproductive process
GOBPID:0060548	negative regulation of cell death
GOBPID:0051050	positive regulation of transport
GOBPID:0030001	metal ion transport
GOBPID:0050878	regulation of body fluid levels
GOBPID:0050801	ion homeostasis
GOBPID:0055082	cellular chemical homeostasis
GOBPID:0035295	tube development

GOBPID:0061061	muscle structure development
GOBPID:0007599	hemostasis
GOBPID:0050817	coagulation
GOBPID:0007596	blood coagulation
GOBPID:0051051	negative regulation of transport
GOBPID:0003013	circulatory system process
GOBPID:0008015	blood circulation
GOBPID:0060537	muscle tissue development
GOBPID:0014706	striated muscle tissue development
GOBPID:0070482	response to oxygen levels
GOBPID:0036293	response to decreased oxygen levels
GOBPID:0001666	response to hypoxia
GOBPID:0050795	regulation of behavior
GOBPID:0032409	regulation of transporter activity
GOBPID:0022898	regulation of transmembrane transporter activity
GOBPID:0032412	regulation of ion transmembrane transporter activity
GOBPID:0001944	vasculature development
GOBPID:0001568	blood vessel development
GOBPID:0055065	metal ion homeostasis
GOBPID:0048589	developmental growth
GOBPID:0006873	cellular ion homeostasis
GOBPID:0030003	cellular cation homeostasis
GOBPID:0006875	cellular metal ion homeostasis
GOBPID:0007507	heart development
GOBPID:0090066	regulation of anatomical structure size
GOBPID:0010720	positive regulation of cell development
GOBPID:0044057	regulation of system process
GOBPID:0071900	regulation of protein serine/threonine kinase activity
GOBPID:0072511	divalent inorganic cation transport
GOBPID:0070838	divalent metal ion transport
GOBPID:0051962	positive regulation of nervous system development
GOBPID:0048545	response to steroid hormone
GOBPID:0072507	divalent inorganic cation homeostasis
GOBPID:0071407	cellular response to organic cyclic compound
GOBPID:0006816	calcium ion transport
GOBPID:0072503	cellular divalent inorganic cation homeostasis
GOBPID:0055074	calcium ion homeostasis
GOBPID:0001701	in utero embryonic development
GOBPID:0050769	positive regulation of neurogenesis
GOBPID:0006874	cellular calcium ion homeostasis
GOBPID:0007517	muscle organ development
GOBPID:0050900	leukocyte migration
GOBPID:0010959	regulation of metal ion transport
GOBPID:0048638	regulation of developmental growth
GOBPID:0051480	cytosolic calcium ion homeostasis
GOBPID:0044706	multi-multicellular organism process
GOBPID:0043270	positive regulation of ion transport
GOBPID:0060541	respiratory system development
GOBPID:0003007	heart morphogenesis
GOBPID:0051924	regulation of calcium ion transport
GOBPID:0009612	response to mechanical stimulus
GOBPID:0030323	respiratory tube development

GOBPID:0051048	negative regulation of secretion
GOBPID:0030324	lung development
GOBPID:1903531	negative regulation of secretion by cell
GOBPID:0050920	regulation of chemotaxis
GOBPID:0034764	positive regulation of transmembrane transport
GOBPID:0034767	positive regulation of ion transmembrane transport
GOCCID:0031012	extracellular
GOCCID:0005578	proteinaceous
GOMFID:0001071	nucleic acid binding transcription factor activity
GOMFID:0003700	transcription factor activity, sequence-specific DNA binding
GOMFID:0000981	RNA polymerase II transcription factor activity, sequence-specific DNA binding
KEGGID:4060	Cytokine-cytokine receptor interaction
KEGGID:4062	Chemokine signaling pathway
KEGGID:5219	Bladder cancer

Table S3. The source of original gene expression profile data [1–35] GPL570 and GPL17027 are different platform. GSE merge numbers represent different series. MDD, Major Depressive Disorder. GBM, glioblastoma.

Diseases	GPL570	GPL17027
MDD	GSE32280, GSE44593, GSE54565, GSE54566, GSE54567, GSE54568, GSE53987, GSE54571, GSE54572	GSE54570, GSE54575
GBM	GSE570, GSE4290, GSE2485, GSE3185, GSE8692, GSE13276, GSE17027, GSE15824, GSE16011, GSE13041, GSE18150, GSE19578, GSE20736, GSE23806, GSE23935, GSE24244, GSE29796, GSE39223, GSE36785, GSE36245, GSE35493, GSE34824, GSE32876, GSE32374, GSE44841, GSE46531, GSE49822, GSE43378, GSE50161, GSE51062, GSE51395, GSE53733, GSE62802, GSE68848	GSE2485, GSE3185, GSE8692, GSE13276

Supplementary reference

1. Khatua, S.; Peterson, K.M.; Brown, K.M.; Lawlor, C.; Santi, M.R.; Lafleur, B.; Dressman, D.; Stephan, D.A.; Macdonald, T.J. Overexpression of the egfr/fkbp12/hif-2α pathway identified in childhood astrocytomas by angiogenesis gene profiling. *Cancer Research* **2003**, *63*, 1865.
2. Dong, S.; Nutt, C.L.; Betensky, R.A.; Stemmerrachamimov, A.O.; Denko, N.C.; Ligon, K.L.; Rowitch, D.H.; Louis, D.N. Histology-based expression profiling yields novel prognostic markers in human glioblastoma. *Journal of Neuropathology & Experimental Neurology* **2005**, *64*, 948.
3. Liang, W.S.; Maddukuri, A.; Teslovich, T.M.; De, l.F.C.; Agbottah, E.; Dadgar, S.; Kehn, K.; Hautaniemi, S.; Pumfery, A.; Stephan, D.A. Therapeutic targets for hiv-1 infection in the host proteome. *Retrovirology* **2005**, *2*, 20.
4. Sun, L.; Hui, A.M.; Su, Q.; Vortmeyer, A.; Kotliarov, Y.; Pastorino, S.; Passaniti, A.; Menon, J.; Walling, J.; Bailey, R. Neuronal and glioma-derived stem cell factor induces angiogenesis within the brain. *Cancer Cell* **2006**, *9*, 287.
5. Liu, T.; Papagiannakopoulos, T.; Puskar, K.; Qi, S.; Santiago, F.; Clay, W.; Lao, K.; Lee, Y.; Nelson, S.F.; Kornblum, H.I. Detection of a microrna signal in an in vivo expression set of mrnas. *PloS one* **2007**, *2*, e804.
6. Lee, Y.; Scheck, A.C.; Cloughesy, T.F.; Lai, A.; Dong, J.; Farooqi, H.K.; Liau, L.M.; Horvath, S.; Mischel, P.S.; Nelson, S.F. Gene expression analysis of glioblastomas identifies the major molecular basis for the

prognostic benefit of younger age. *Bmc Medical Genomics* **2008**, *1*, 52.
7. Gravendeel, L.A.; Kouwenhoven, M.C.; Gevaert, O.; de Rooi, J.J.; Stubbs, A.P.; Duijm, J.E.; Daemen, A.; Bleeker, F.E.; Bralten, L.B.; Kloosterhof, N.K. Intrinsic gene expression profiles of gliomas are a better predictor of survival than histology. *Cancer Research* **2009**, *69*, 9065.
8. Madhavan, S.; Zenklusen, J.C.; Kotliarov, Y.; Sahni, H.; Fine, H.A.; Buetow, K. Rembrandt: Helping personalized medicine become a reality through integrative translational research. *Molecular Cancer Research Mcr* **2009**, *7*, 157.
9. Raluca, T.; Mahé, M.M.; Laurianne, V.L.; Jean, L.; Isabelle, G.; Rémi, H.; Michel, N. Regulation of intestinal epithelial cells transcriptome by enteric glial cells: Impact on intestinal epithelial barrier functions. *Bmc Genomics* **2009**, *10*, 507.
10. Suvà, M.L.; Riggi, N.; Janiszewska, M.; Radovanovic, I.; Provero, P.; Stehle, J.C.; Baumer, K.; Le, B.M.; Marino, D.; Cironi, L. Ezh2 is essential for glioblastoma cancer stem cell maintenance. *Cancer Research* **2009**, *69*, 9211-9218.
11. J, A.; A, S.-B.; A, G.-J.; L, R.; G, F.; MA, C.; RM, P.-S.; I, B.; E, M.-S.; L, P., *et al.* Tgf-β receptor inhibitors target the cd44 high /id1 high glioma-initiating cell population in human glioblastoma. *Cancer Cell* **2010**, *18*, 655-668.
12. Suzuki, K.; Iwata, Y.; Matsuzaki, H.; Anitha, A.; Suda, S.; Iwata, K.; Shinmura, C.; Kameno, Y.; Tsuchiya, K.J.; Nakamura, K. Reduced expression of apolipoprotein e receptor type 2 in peripheral blood lymphocytes from patients with major depressive disorder. *Progress in Neuro-Psychopharmacology and Biological Psychiatry* **2010**, *34*, 1007-1010.
13. Wang, R.; Chadalavada, K.; Wilshire, J.; Kowalik, U.; Hovinga, K.E.; Geber, A.; Fligelman, B.; Leversha, M.; Brennan, C.; Tabar, V. Glioblastoma stem-like cells give rise to tumour endothelium. *Nature* **2010**, *468*, 829-833.
14. Bielen, A.; Perryman, L.; Box, G.M.; Valenti, M.; Brandon, A.D.H.; Martins, V.; Jury, A.; Popov, S.; Gowan, S.; Jeay, S. Enhanced efficacy of igf1r inhibition in paediatric glioblastoma by combinatorial targeting of pdgfrα/β. *Molecular Cancer Therapeutics* **2011**, *10*, 1407.
15. Grzmil, M.; Jr, M.P.; Lino, M.M.; Merlo, A.; Frank, S.; Wang, Y.; Moncayo, G.; Hemmings, B.A. Map kinase-interacting kinase 1 regulates smad2-dependent tgf-β signaling pathway in human glioblastoma. *Cancer Research* **2011**, *71*, 2392.
16. Nogueira, L.; Ruiz-Onta, P.; ǀon; Vazquez-Barquero, A.; Lafarga, M.; Berciano, M.T.; Aldaz, B.; Grande, L.; Casafont, I.; Segura, V., *et al.* Blockade of the nfκb pathway drives differentiating glioblastoma-initiating cells into senescence both in vitro and in vivo. *Oncogene* **2011**, *30*, 3537.
17. Nagano, A.J.; Sato, Y.; Mihara, M.; Antonio, B.A.; Motoyama, R.; Itoh, H.; Nagamura, Y.; Izawa, T. Deciphering and prediction of transcriptome dynamics under fluctuating field conditions. *Cell* **2012**, *151*, 1358.
18. Schwartzentruber, J.; Korshunov, A.; Liu, X.Y.; Jones, D.T.W.; Pfaff, E.; Jacob, K.; Sturm, D.; Fontebasso, A.M.; Quang, D.A.K.; Tnjes, M. Driver mutations in histone h3.3 and chromatin remodelling genes in paediatric glioblastoma. *Nature* **2012**, *482*, 226.
19. Setty, M.; Helmy, K.; Khan, A.A.; Silber, J.; Arvey, A.; Neezen, F.; Agius, P.; Huse, J.T.; Holland, E.C.; Leslie, C.S. Inferring transcriptional and microrna-mediated regulatory programs in glioblastoma. *Molecular Systems Biology* **2012**, *8*, 605-605.
20. Sturm, D.; Witt, H.; Hovestadt, V.; Khuongquang, D.A.; Jones, D.T.; Konermann, C.; Pfaff, E.; Tönjes, M.; Sill, M.; Bender, S. Hotspot mutations in h3f3a and idh1 define distinct epigenetic and biological subgroups of glioblastoma. *Cancer Cell* **2012**, *22*, 425.
21. Yi, Z.; Li, Z.; Yu, S.; Wang, L.; Yuan, C.; Hong, W.; Shi, T.; Fang, Y. Blood-based gene expression profiles models for classification of subsyndromal symptomatic depression and major depressive disorder. *PloS one* **2012**, *7*, e31283.
22. Aldaz, B.; Sagardoy, A.; Nogueira, L.; Guruceaga, E.; Grande, L.; Huse, J.T.; Aznar, M.A.; Díezvalle, R.; Tejadasolís, S.; Alonso, M.M. Involvement of mirnas in the differentiation of human glioblastoma multiforme stem-like cells. *PloS one* **2013**, *8*, e77098.

23. Annunziato, M.; Nathalie, S.; Pasquale, D.B.; Daniela, O.; Gigliola, S.; Gina, L.; Ludovica, P.B.; Giovanni, S.; Marcella, Z.; Libero, L. Gene expression profile of glioblastoma peritumoral tissue: An ex vivo study. *PloS one* **2013**, *8*, e57145.
24. Auvergne, R.M.; Sim, F.J.; Wang, S.; Chandlermilitello, D.; Burch, J.; Al, F.Y.; Davis, D.; Benraiss, A.; Walter, K.; Achanta, P. Transcriptional differences between normal and glioma-derived glial progenitor cells identify a core set of dysregulated genes. *Cell Reports* **2013**, *3*, 2127.
25. Bender, S.; Tang, Y.; Lindroth, A.M.; Hovestadt, V.; Jones, D.T.; Kool, M.; Zapatka, M.; Northcott, P.A.; Sturm, D.; Wang, W. Reduced h3k27me3 and DNA hypomethylation are major drivers of gene expression in k27m mutant pediatric high-grade gliomas. *Cancer Cell* **2013**, *24*, 660-672.
26. Birks, D.K.; Donson, A.M.; Patel, P.R.; Sufit, A.; Algar, E.M.; Dunham, C.; Kleinschmidtdemasters, B.K.; Handler, M.H.; Vibhakar, R.; Foreman, N.K. Pediatric rhabdoid tumors of kidney and brain show many differences in gene expression but share dysregulation of cell cycle and epigenetic effector genes. *Pediatric Blood & Cancer* **2013**, *60*, 1095-1102.
27. Griesinger, A.M.; Birks, D.K.; Donson, A.M.; Amani, V.; Hoffman, L.M.; Waziri, A.; Wang, M.; Handler, M.H.; Foreman, N.K. Characterization of distinct immunophenotypes across pediatric brain tumor types. *Journal of Immunology* **2013**, *191*, 4880-4888.
28. Kawaguchi, A.; Yajima, N.; Tsuchiya, N.; Homma, J.; Sano, M.; Natsumeda, M.; Takahashi, H.; Fujii, Y.; Kakuma, T.; Yamanaka, R. Gene expression signature-based prognostic risk score in patients with glioblastoma. *Cancer Science* **2013**, *104*, 1205–1210.
29. Masiero, M.; Simões, F.C.; Han, H.D.; Snell, C.; Peterkin, T.; Bridges, E.; Mangala, L.S.; Wu, Y.Y.; Pradeep, S.; Li, D. A core human primary tumor angiogenesis signature identifies the endothelial orphan receptor eltd1 as a key regulator of angiogenesis. *Cancer Cell* **2013**, *24*, 229-241.
30. Ye, F.; Zhang, Y.; Liu, Y.; Yamada, K.; Tso, J.L.; Menjivar, J.C.; Tian, J.Y.; Yong, W.H.; Schaue, D.; Mischel, P.S. Protective properties of radio-chemoresistant glioblastoma stem cell clones are associated with metabolic adaptation to reduced glucose dependence. *PloS one* **2013**, *8*, e80397.
31. Chang, L.C.; Jamain, S.; Lin, C.W.; Dan, R.; Tseng, G.C.; Sibille, E. A conserved bdnf, glutamate- and gaba-enriched gene module related to human depression identified by coexpression meta-analysis and DNA variant genome-wide association studies. *PloS one* **2014**, *9*, e90980.
32. Reifenberger, G.; Weber, R.G.; Riehmer, V.; Kaulich, K.; Willscher, E.; Wirth, H.; Gietzelt, J.; Hentschel, B.; Westphal, M.; Simon, M. Molecular characterization of long-term survivors of glioblastoma using genome- and transcriptome-wide profiling. *International Journal of Cancer* **2014**, *135*, 1822.
33. Zamykal, M.; Martens, T.; Matschke, J.; Günther, H.S.; Kathagen, A.; Schulte, A.; Peters, R.; Westphal, M.; Lamszus, K. Inhibition of intracerebral glioblastoma growth by targeting the insulin-like growth factor 1 receptor involves different context-dependent mechanisms. *Neuro-oncology* **2014**, *17*, 1076.
34. Chong, Y.K.; Sandanaraj, E.; Koh, L.W.H.; Thangaveloo, M.; Tan, M.S.Y.; Koh, G.R.H.; Tan, B.T.; Lim, G.G.Y.; Holbrook, J.D.; Kon, O.L. St3gal1-associated transcriptomic program in glioblastoma tumor growth, invasion, and prognosis. *Journal of the National Cancer Institute* **2015**, *108*.
35. Walsh, A.M.; Kapoor, G.S.; Buonato, J.M.; Mathew, L.K.; Bi, Y.; Davuluri, R.V.; Martinezlage, M.; Simon, M.C.; O'Rourke, D.M.; Lazzara, M.J. Sprouty2 drives drug resistance and proliferation in glioblastoma. *Molecular Cancer Research Mcr* **2015**, *13*, 1227.
